# New Method of Operating for Extirpation of the Testicle

**Published:** 1850-04

**Authors:** 


### SURGERY.

*New Method of Operating for Extirpation of the Testicle.*—The Gazette des Hopitaux mentions a new mode of incision in extirpation of the testicle, introduced by M. Jobert, surgeon to the Hotel Dieu. Instead of making simply a longitudinal or an elliptical incision on the anterior part of the testicle, M. Jobert makes a shell-like or valvular opening, by carrying his incision in a semi-circular form along the lower convexity of the organ. The usual method has been found objectionable, because a deep cavity is left after the removal of the testicle, in which fluids may accumulate, and this circumstance seldom allows of union by first intention. Aumont had formerly proposed to attack the tumor inferiorly and posteriorly, both to prevent accumulation and avoid the anterior cicatrix, but M. Jobert prefers the above-mentioned method, and proceeds thus: "The bistoury, held like a pen, is plunged into the inferior part of the inguinal canal, and carried, from above downwards, along the external and anterior side of the tumor, as far as its basis, where the instrument is made to sweep round towards the internal side and ascend in the direction of the ring, without, however, running so far as that point, but stopping by the side of the penis. The lips of the wound separate of themselves, and the bistoury needs only be made to pass under the anterior flap, and then under the posterior, in order to isolate the whole tumor with an astonishing rapidity. The vessels may be tied as they are being divided. By this process, the two flaps will be brought in contact without the least effort, or leaving any cavity; and the surgeon has no occasion to fear the infiltration of any liquid. The favorable circumstances will be conducive to union by first intention, the hope of which had almost been abandoned by surgeons in cases of castration. The threads, instead of being placed in the middle of the scrotum, as is the case in the ordinary method of operating, are placed along a semi-circle, and act as conductors to the serosity which commonly forms in this region after the operation. This incision was applied in a case of encephaloid tumor of the testicle, on the 23d of November last; and the wound was secured by the twisted suture. Compresses of cold water were applied; on the 24th there was no fever and very little swelling; on the 25th no fever, and on the 26th half of the pins were removed. On the 15th of December, the wound had been for some days completely cicatrized. This is not quite union by first intention, but the ligatures of the cord may have prevented that result.—*Lond. Lancet.*

